# Endoscopic ultrasound guided vascular intervention with digital subtraction angiography for isolated gastric varices: report of a video case

**DOI:** 10.1055/a-2792-9487

**Published:** 2026-02-27

**Authors:** Kazunori Nagashima, Tomoya Sakamoto, Ayako Nagasaki, Kengo Matsumoto, Tsunehiro Suzuki, Manabu Misu, Atsushi Irisawa

**Affiliations:** 1 12756 Department of Gastroenterology, Dokkyo Medical University School of Medicine, Mibu, Japan; 2 46624 Japanese Red Cross Ashikaga Hospital, Ashikaga, Japan

In recent years, endoscopic ultrasound-guided vascular intervention (EUS-VI) has been developed for varices 1 2 3 . Some facilities often perform EUS-VI without using fluoroscopy. However, we infer the importance of using fluoroscopy and evaluating blood flow for safe EUS-VI 4 5 . Therefore, we perform EUS-VI using not only standard fluoroscopy but also digital subtraction angiography (DSA) to evaluate blood flow during treatment. This video case is the first to present EUS-VI with DSA for gastric varices.

**Fig. 1:**
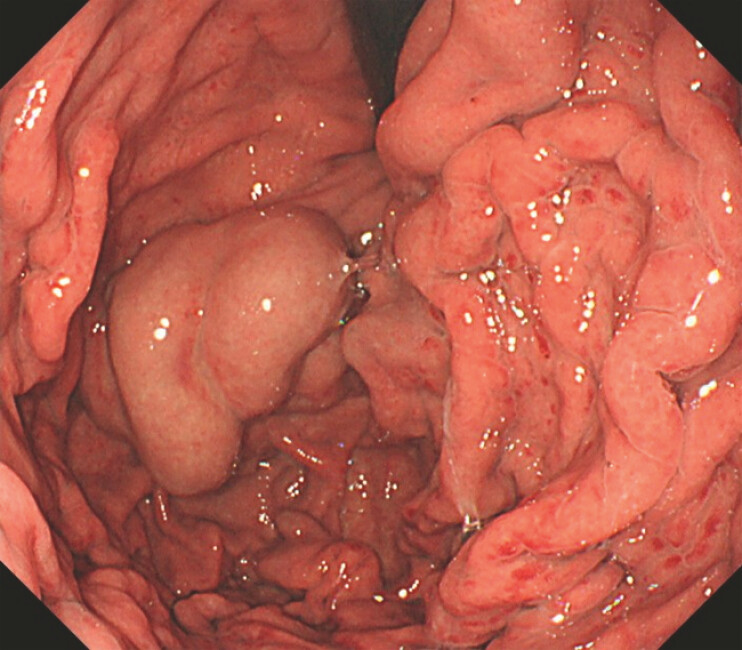
The varices were large. They showed strong development.

**Fig. 2:**
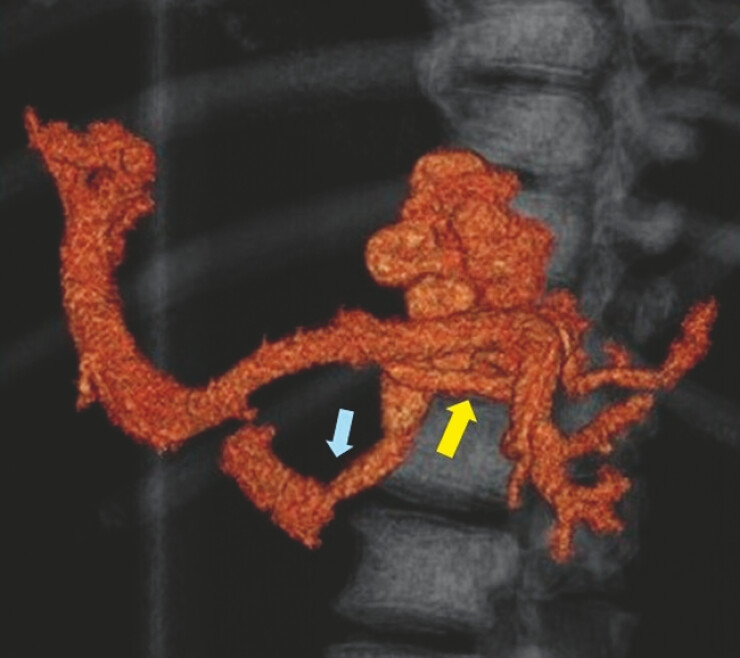
3D-CT revealed the hemodynamics of varices fed from the posterior gastric vein (yellow arrow) through the gastric varices to the gastrorenal shunt (blue arrow). 3D-CT, three-dimensional contrast-enhanced computed tomography.

**Fig. 3:**
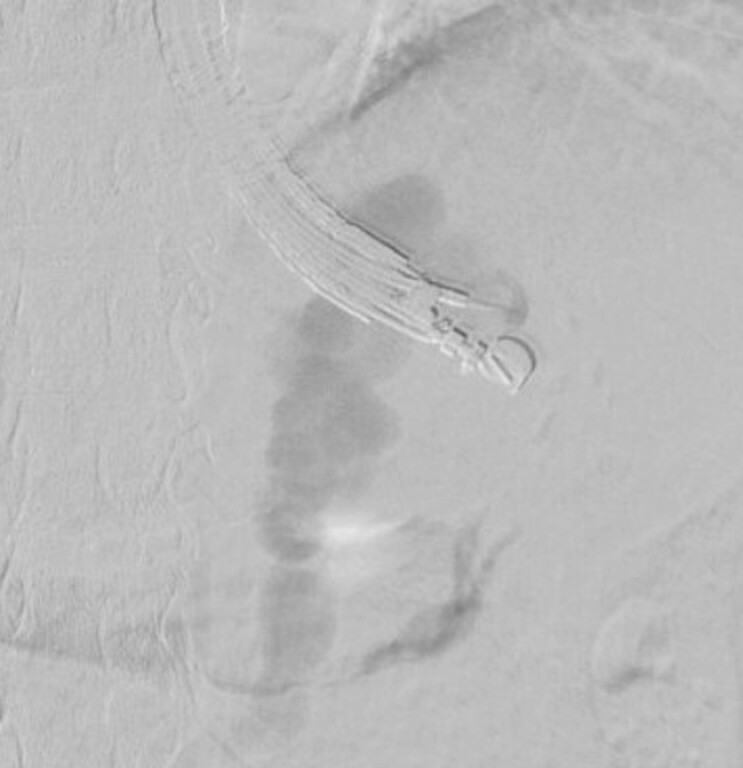
DAS showed drainage flow (gastrorenal shunt). DAS, digital subtraction angiography.

**Fig. 4:**
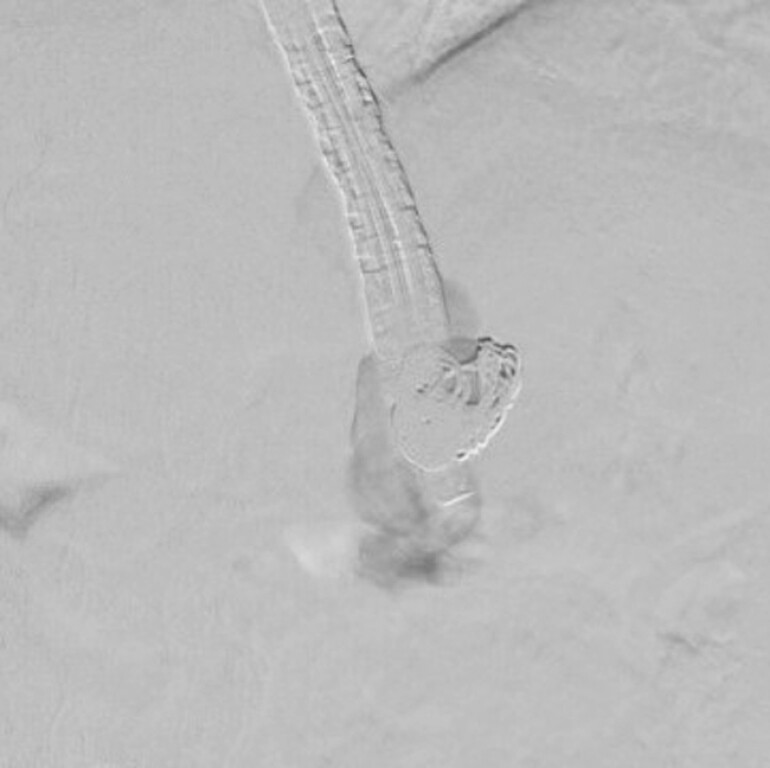
Some coils were placed. A sclerosant (EO) and a cyanoacrylate (CA) were injected into the varices.

**Fig. 5:**
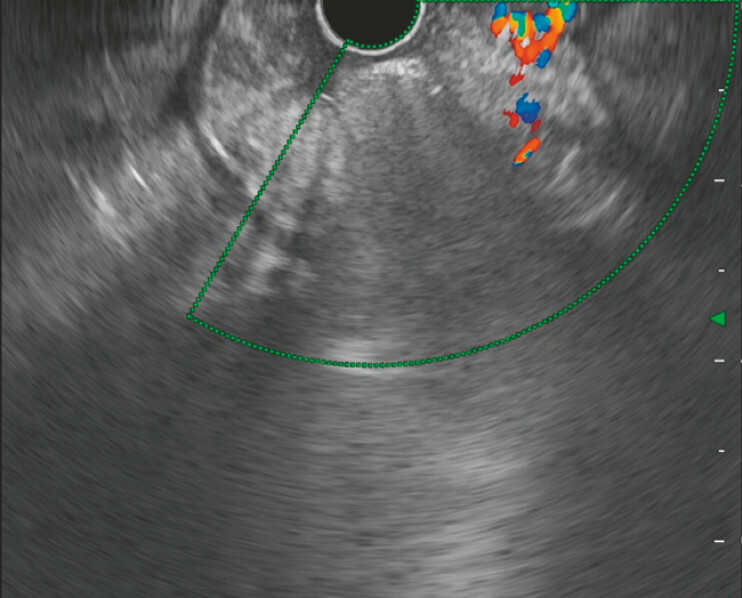
EUS revealed that coils were placed and that the varix flow had disappeared. EUS, endoscopic ultrasound.

This video shows a typical case ( Video 1 ). The patient, a 55-year-old man, had alcoholic cirrhosis and giant isolated gastric varices ( Fig. 1 ). Three-dimensional contrast-enhanced computed tomography (3D-CT) showed the hemodynamics of the varices, which were fed from the posterior gastric vein through the gastric varices to the gastrorenal shunt ( Fig. 2 ). After the varices were punctured using a 19G FNA needle (EZ shot3 plus; Olympus Corp., Tokyo, Japan), DSA was performed. The hemodynamics including a drainage vein (gastrorenal vein) was shown clearly by DSA ( Fig. 3 ). After a 0.035-inch hydrocoil (Azur; Terumo Corp. Tokyo, Japan) was placed, a sclerosant (EO) and a cyanoacrylate (CA) were injected into the varices for the blood supply route ( Fig. 4 ). Thereafter, the blood flow ceased ( Fig. 5 ). One week later, it was confirmed that the complete blood flow had been stopped with only one session of treatment.

This report presents the EUS-guided vascular intervention with DSA for gastric varices. This EUS-VI with DSA is useful and safe for vascular procedures of EUS-VI. DAS, digital subtraction angiography; EUS, endoscopic ultrasound; EUS-VI, endoscopic ultrasound-guided vascular intervention.Video 1

We usually perform EUS-VI with DSA in the catheterization laboratory. The method can evaluate blood flow clearly even in cases of fast blood flow. This capability is important to ensure treatment safety. DSA information enables us to determine the coil size, the coil deployment position, the EO and CA injection amounts, and the injection timing. We consider that EUS-VI with DSA make the procedure safer. Furthermore, it is useful not only for varices but also for various other vascular procedures of EUS-VI.

Endoscopy_UCTN_Code_TTT_1AS_2AG
